# S7-5 How scared are you? A scoping of review of the consequences of eco-anxiety on physical activity

**DOI:** 10.1093/eurpub/ckad133.037

**Published:** 2023-09-11

**Authors:** Thibaut Derigny, François Potdevin, Joseph Gandrieau, Léa Mekkaoui, Christophe Schnitzler

**Affiliations:** Univ. Lille, Univ. Artois, Univ. Littoral Côte d’Opale, Laboratoire URePSSS – Unité de Recherche Pluridisciplinaire Sport Santé Société – ULR 7369, F-59000 Lille, France; Univ. Lille, Univ. Artois, Univ. Littoral Côte d’Opale, Laboratoire URePSSS – Unité de Recherche Pluridisciplinaire Sport Santé Société – ULR 7369, F-59000 Lille, France; 1 Univ. Lille, Univ. Artois, Univ. Littoral Côte d’Opale, Laboratoire URePSSS – Unité de Recherche Pluridisciplinaire Sport Santé Société. 2. Université Côte d’Azur, Laboratoire LAMHESS – Laboratoire Motricité Humaine Expertise Sport Santé; Univ. Lille, Univ. Artois, Univ. Littoral Côte d’Opale, Laboratoire URePSSS – Unité de Recherche Pluridisciplinaire Sport Santé Société – ULR 7369, F-59000 Lille, France; Faculté des Sciences du Sport, Laboratoire E3S – Éducation Sport et Sciences Sociales – ULR1342, Université de Strasbourg, France

## Abstract

**Purpose:**

Physical activity (PA) is a good way to improve people’s health, and when used as a means for active transportation, it reduces up to ten times GES emissions as compared to cars. Promoting active transportation potentially impacts positively the United Nations’ Sustainable Development Goal 3 (‘good health’), 11 (‘sustainable cities and communities’) and 13 (‘climate action’). However, the prevalence of fears due to environmental anxiety (‘eco-anxiety’) has grown significantly over the last decade. Eco-anxiety are feeling of fear, worry, or concern about the state of the environment and the potential consequences of climate change and other environmental issues. It can be caused by a variety of factors, including exposure to degraded and polluted environment, extreme climatic events, but also media coverage of environmental disasters, a lack of government action on environmental issues, and a sense of helplessness or powerlessness to make a difference. Nevertheless, to our knowledge, the effect of eco-anxiety on PA has not been documented. Our research questions are: (1) what are the effects of eco-anxiety on PA? (2) to what extend is it reducing active transportation? (3) can the promotion of active transportation helps fight the consequences of eco-anxiety? (4) what evidence gaps require further research for promotion and public policies?

**Methods:**

We undertake a comprehensive scoping review. We search in eleven main databases studies that explicitly question the relationship between PA and CO2 carbon emissions. The evidence from eligible reviews will be extracted and mapped according to the format Participant, Context, Concept. We will classify the overall findings as positive, negative, or inconclusive, in their relation between perception and PA practices.

**Results:**

Data collection is currently in progress. We hypothesise that eco-anxiety has negative effects on PA, especially on active transportation, but that a promotion by publics policies which considers this phenomenon can achieve the dual objective of individual health / GES reduction.

**Conclusions:**

Eco-anxiety can lead to paradoxical effects that should be better understood, so that public policies should orient their promotion of strategies to reduce CO2 emissions by using active transport, while avoiding their deleterious effects.

**Support/Funding Source:**

no funding.

